# Pancreatic Adverse Events Associated With Immune Checkpoint Inhibitors: A Large-Scale Pharmacovigilance Analysis

**DOI:** 10.3389/fphar.2022.817662

**Published:** 2022-04-01

**Authors:** Yue Zhang, Yisheng Fang, Jianhua Wu, Genjie Huang, Jianping Bin, Yulin Liao, Min Shi, Wangjun Liao, Na Huang

**Affiliations:** ^1^ Department of Oncology, Nanfang Hospital, Southern Medical University, Guangzhou, China; ^2^ Department of Cardiology, Nanfang Hospital, Southern Medical University, Guangzhou, China

**Keywords:** immune checkpoint inhibitors, PD-1/PD-L1, CTLA-4, combination therapy, pancreatic adverse events, pancreatitis, diabetes mellitus, FAERS

## Abstract

**Backgrounds:** Immune checkpoint inhibitors (ICIs) are considered cornerstones of oncology treatment with durable anti-tumor efficacy, but the increasing use of ICIs is associated with the risk of developing immune-related adverse events (irAEs). Although ICI-associated pancreatic adverse events (AEs) have been reported in patients treated with ICIs, the clinical features and spectrum of pancreatic AEs are still not well-defined. Therefore, this study aimed to identify the association between pancreatic AEs and ICIs treatments and to characterize the main features of ICI-related pancreatic injury (ICIPI) based on the Food and Drug Administration Adverse Event Reporting System (FAERS) database. Methods: Data from the first quarter of 2015 to the first quarter of 2021 in the database were extracted to conduct a disproportionality analysis. The selection of AEs related to the pancreas relied on previous studies and preferred terms from the Medical Dictionary for Regulatory Activities. Two main disproportionality analyses—the reporting odds ratio (ROR) and information component (IC)—were used to evaluate potential associations between ICIs and pancreatic AEs. Results: In total, 2,364 cases of pancreatic AEs in response to ICIs were extracted from the FAERS database, of which, 647 were identified as ICI-associated pancreatitis and 1,293 were identified as ICI-associated diabetes mellitus. Generally, significant signals can be detected between pancreatic AEs and all ICIs treatments (ROR_025_ = 3.30, IC_025_ = 1.71). For monotherapy, the strongest signal associated with pancreatitis was reported for anti-PD-L1 (ROR_025_ = 1.75, IC_025_ = 0.76), whereas that with diabetes mellitus was reported for anti-PD-1 (ROR_025_ = 6.39, IC_025_ = 2.66). Compared with monotherapy, combination therapy showed stronger associations with both ICI-associated pancreatitis (ROR_025_ = 2.35, IC_025_ = 1.20 *vs*. ROR_025_ = 1.52, IC_025_ = 0.59) and ICI-associated diabetes mellitus (ROR_025_ = 9.53, IC_025_ = 3.23 *vs*. ROR_025_ = 5.63, IC_025_ = 2.48), but lower fatality proportion. Conclusions: ICIs were significantly associated with the over-reporting frequency of pancreatic AEs, in which combination therapy posed a higher reporting frequency. Therefore, patients should be informed of these potential toxicities before ICIs medications are administered.

## Introduction

Immune checkpoint inhibitors (ICIs) that block cytotoxic T lymphocyte antigen-4 (CTLA-4), programmed cell death protein (PD-1), and its ligand PD-L1 have been considered among the most important developments in oncology in recent years (Martins et al., 2019). By “releasing the brakes” on anti-tumor immune effects and promoting T-cell-mediated immune responses (Johnson et al., 2018; Khan and Gerber, 2020), ICIs have shown remarkable benefits in a wide array of cancer types, including melanoma, non-small cell lung cancer, renal cell carcinoma, and head and neck cancers (Vaddepally et al., 2020).

Apart from impressive anti-tumor efficacy, enhancement of immune responses by ICIs may also promote T lymphocyte activity systematically, thereby facilitating a range of autoimmune toxicity potentially against any organ, which is often referred to as immune-related adverse events (irAEs) (Lyon et al., 2018; Postow et al., 2018). The most commonly involved organs are those in the gastrointestinal, dermatologic, hepatic, and endocrine systems (Postow et al., 2018), most of which have been studied extensively through pharmacovigilance analyses (Vozy et al., 2019; Zhai et al., 2019; Hu et al., 2020; Chen et al., 2021b).

With the widespread clinical use of ICIs, an increasing number of cases of relatively uncommon irAEs have been reported, including ICI-related pancreatic injury (ICIPI) (Abu-Sbeih et al., 2019; Porcu et al., 2020; Liu et al., 2021). The findings from a meta-analysis showed that the incidence of ICIPI was 0.9–3% for anti-CTLA-4 monotherapy, 0.5–1.6% for anti-PD-1 monotherapy, and 1.2–2.1% for combination therapy of anti-CTLA-4 and anti-PD-1 (Su et al., 2018). Some pharmacovigilance studies generally describe ICI-related AEs in the gastrointestinal system, including pancreatitis (Reese et al., 2020; Bai et al., 2021). However, knowledge is still scarce about the detailed safety profile of pancreatic AEs following various ICIs in real-world clinical practice as no pancreatic AEs other than pancreatitis and autoimmune pancreatitis is included in the existing pharmacovigilance studies. In addition, the association between different ICIs and pancreatitis is controversial. Some previous studies showed that the risk of pancreatitis was higher with anti-CTLA-4 than that with anti-PD-1 or anti-PD-L1 (Su et al., 2018; Bai et al., 2021), while another study found that anti-PD-L1 versus anti-PD-1 or anti-CTLA-4 was associated with a slightly increased connection of pancreatitis (Reese et al., 2020). Moreover, increased T lymphocytes may attack pancreatic cells such as islet β-cells and acinar cells, which will not only lead to endocrine disorders but also cause damage to exocrine function. It may eventually result in irreversible lesions and potentially life-threatening conditions if not promptly recognized and treated (Stamatouli et al., 2018; Abu-Sbeih et al., 2019; Quandt et al., 2020; Liu et al., 2021). Therefore, it is necessary to determine whether there exists a connection between different ICIs and pancreatic AEs and to evaluate the detailed safety profile of ICIPI for further prevention and management. The United States Food and Drug Administration Adverse Event Reporting System (FAERS) serves as a publicly accessible repository of spontaneous adverse drug event reports submitted to the FDA by healthcare professionals, individual patients, pharmacists, manufacturers, and other sources (Brinker et al., 2013). The database contains millions of reports that not only cover the entire American population, but also include reports from other countries, which is important for investigating a previously unknown drug reaction (Alshammari and AlMutairi, 2015).

Herein, to address the gap in knowledge, this study aimed to evaluate the characteristics and associations between pancreatic AEs and ICIs treatments by using the FAERS database and to determine the time to onset, hospitalization and fatality proportion of pancreatic adverse events following different ICIs treatments.

## Materials and Methods

### Data Source

Data from a real-world retrospective study were extracted from the FAERS database dated from the first quarter (Q1) of 2015 to the Q1 of 2021. Adverse event (AE) information in the database contains seven types of datasets: DEMO file (patient demographic and administrative information), DRUG file (drug information), REACTION file (AEs coded by MedDRA terminology), OUTCOME file (patient outcomes), RPSR file (report sources), THERAPY file (therapy start dates and end dates for reported medications), and INDICATIONS file (the indications for the reported drugs) (Chen et al., 2020). We selected data considering important variables such as age, sex, drug name, preferred terms (PTs) for adverse drug reactions, outcomes, and indications from the FAERS database. Duplicate reports inevitably exist in spontaneous reporting data, and to remove duplicates, we employed a widely used method called variable matching, namely, two reports are regarded as duplicate reports if the key variables are the same (Tregunno et al., 2014). PRIMARY ID, CASE ID, and FDA_DT were selected as the key matching variables. Then, we performed the procedure for data quality by choosing the latest FDA_DT when the CASE IDs were equivalent and chose the higher PRIMARY ID when the CASE ID and FDA_DT were the same, as recommended by the U.S. Food and Drug Administration (Hu et al., 2020; Chen et al., 2021a; Chen et al., 2021b).

### Adverse Event and Drug Identification

As a result of limited information about ICIPI in published studies, to the best of our knowledge, there is no gold standard for classifying and selecting PTs for ICIPI. According to previous studies, pancreatic AEs that occurred in cancer patients treated with ICIs are collectively described as ICIPI including pancreatitis, asymptomatic pancreatic enzyme elevation, hyperglycemia, diabetes mellitus, and exocrine pancreatic insufficiency (Abu-Sbeih et al., 2019; Porcu et al., 2020; Liu et al., 2021). ICI-associated diabetes mellitus (ICI-DM) is defined as new-onset insulin-dependent diabetes, characterized by an acute attack of dramatic hyperglycemia with the destruction of beta cells and severe insulin deficiency (Stamatouli et al., 2018; de Filette et al., 2019; Quandt et al., 2020). A retrospective study has reported that lipase elevation is mainly caused by extra-pancreatic AEs such as colitis rather than pancreatitis (Grimmelmann et al., 2021). Considering that the relevance of lipase elevation with ICI-associated pancreatitis (ICI-P) remains controversial (Friedman et al., 2017; Grimmelmann et al., 2021), pancreatic enzyme elevation was excluded from the study. Based on previous reports (Raschi et al., 2013; Friedman et al., 2017; Eshet et al., 2018; Stamatouli et al., 2018; Wright et al., 2018; Abu-Sbeih et al., 2019; de Filette et al., 2019; Liu et al., 2020; Porcu et al., 2020; Quandt et al., 2020; Grimmelmann et al., 2021; Liu et al., 2021), the PTs of ICIPI selected from the Medical Dictionary for Regulatory Activities (MedDRA) and included in this study are provided in Supplementary Table S1.

Both brand names and generic names were used to identify ICI-associated records, owing to the permission for the registration of drug names arbitrarily in FAERS (Zhai et al., 2019). Therefore, drugs in this study were identified as follows: ipilimumab/Yervoy, cemiplimab/Libtayo, nivolumab/Opdivo, pembrolizumab/Keytruda, atezolizumab/Tecentriq, avelumab/Bavencio, and durvalumab/Imfinzi. The role of the drug in the emergence of AEs was categorized into four types: primary suspect (PS), secondary suspect (SS), concomitant (C), and interacting (I). To obtain better signal intensity, reports were restricted to those in which drugs were coded as “PS” in this study.

### Data Mining

Disproportionality analysis is widely applied to compare the proportion of selected AEs caused by the target drugs with the proportion of the same AEs in the full database. In our study, all drugs in the database were selected as comparisons for the disproportionality approach. However, the disproportionality signals will be inaccurate if the ICIs are only compared with all drugs and not with drugs used for similar indications (Raschi et al., 2020; Jedlowski et al., 2021). Therefore, analyses were performed under the following methods to further assess the pancreatic AEs of ICIs: selecting a dataset where only anticancer therapies (mainly chemotherapy) are represented as well as using relevant reports as comparisons (Hu et al., 2021), which can be seen in the supplementary material (Supplementary Table S2). The reporting odds ratio (ROR) and information component (IC) are widely used to calculate disproportionality to assess potential associations between targeted drugs and selected AEs (Raschi et al., 2018). The statistical shrinkage transformation was considered for consistency and robustness. ROR and IC following shrinkage transformation can be estimated as follows (Norén et al., 2013; Zhai et al., 2019; Chen et al., 2021a):

$$\text{ROR }=\;\frac{{\text{N}}_{\text{observed}}\;+\;0.5}{{\text{N}}_{\text{expected}}\;+\;0.5}$$

$$\text{IC }={\text{ log}}_{2}\frac{{\text{N}}_{\text{observed}}\;+\;0.5}{{\text{N}}_{\text{expected}}\;+\;0.5}$$

$${\text{N}}_{\text{expected}}=\;\frac{{\text{N}}_{\text{drug}}\;\times {\text{ N}}_{\text{event}}}{{\text{N}}_{\text{total}}}$$

N_observed_: the number of records observed for the selected AEs.

N_expected_: the number of records expected for the selected AEs.

N_drug_: the total number of records for the targeted drug with AEs excluded from consideration.

N_event_: the total number of records for the selected AEs regardless of drugs.

N_total_: the total number of records for all the drugs in the database.

ROR was defined as a significant signal if ROR_025_ (lower end of the 95% confidence interval of ROR) exceeded one, with at least three records. For IC, an IC_025_ (lower end of the 95% confidence interval of IC) of more than zero was deemed significantly different. The formulas used to calculate ROR_025_ and IC_025_ are as follows (Norén et al., 2013; Ma et al., 2021):

$${\text{ROR}}_{025}\;={\text{ e}}^{\text{ln}\left(\text{ROR}\right)\;-\;1.96\sqrt{\frac{1}{\text{a}}\;+\;\frac{1}{\text{b}}\;+\;\frac{1}{\text{c}}\;+\;\frac{1}{\text{d}}}}$$

$${\text{IC}}_{025}\;=\text{ IC }-\;3.3\;\times \;{\left({\text{N}}_{\text{observed}}\;+\;0.5\right)}^{-0.5}-2\;\times \;{\left({\text{N}}_{\text{observed}}\;+\;0.5\right)}^{-1.5}$$

Both ROR_025_ and IC_025_ were calculated to assess the association between all pancreatic AEs and different ICIs therapies, while IC_025_, which indicates the signal intensity, was calculated in the spectrum of pancreatic AEs (Chen et al., 2021a). The time to onset (TTO) of ICIPI was identified as the time span between the START_DT (start date of the administration of ICIs) and EVENT_DT (the date of the AE onset). Prior to calculating the onset time, reports were excluded when the START_DT was later than the EVENT_DT or when the report lacked a START_DT or EVENT_DT. We also calculated the fatality proportion in patients with ICIPI, which was determined as the proportion of fatal events to the total events of ICIPI.

### Statistical Analysis

We conducted descriptive analyses to summarize the characteristics of all reports on ICIPI. The time from the beginning of the treatment to the happening of adverse events was compared between different ICIs using nonparametric tests, among which, the Mann-Whitney test was suitable for dichotomous variables and the Kruskal-Wallis test was suitable for more than two independent samples. We used Pearson’s chi-square test or Fisher’s exact test to compare fatality proportion and hospitalization between different ICIs. *p* < 0.05, with 95% confidence intervals, was indicative of statistical significance. The results were analyzed using SPSS version 20.0 software.

## Results

### Descriptive Analysis

A total of 15,118,019 reports of AEs were extracted from the FAERS database, of which, 204,702 reports were induced by ICIs and 50,721 reports were related to pancreatic AEs. In total, 2,364 cases of pancreatic AEs in response to ICIs treatments were identified, of which, 647 were reported as ICI-P and 1,293 were reported as ICI-DM, accounting for the majority of ICIPI cases. The demographic and clinical characteristics of the patients are summarized in Table 1. Most of the ICI-associated pancreatic AEs were from cases in America (35.36%), followed by cases in Japan (29.74%) and France (7.02%). ICIPI cases were most frequently reported in melanoma (30.67%). As shown in Table 1, the number of AEs following ICIs treatments has increased gradually, and pancreatic AEs were no exception. Although male patients accounted for a larger proportion of patients with ICIPI than female patients (58.72 *vs*. 35.15%), patients with ICIPI had a similar reporting frequency between male and female patients (1.16 *vs*. 1.16%, χ^2^ = 0.004, *p* = 0.950). In addition, no significant difference was noted in the reporting frequency between male and female patients with ICI-P (0.30 *vs*. 0.33%, χ^2^ = 0.690, *p* = 0.406). In patients with ICI-DM, male patients also accounted for a larger proportion (59.40%) but with a reporting frequency nearly similar to that of female patients (0.64 *vs*. 0.63%, χ^2^ = 0.089, *p* = 0.765).

**TABLE 1:** Clinical characteristics of patients with ICI-associated pancreatic AEs.

Characteristics	Reports of ICIs (204,702)	Reports of ICIPI (2,364)	Reports of ICI-P (647)	Reports of ICI-DM (1,293)
Gender				
Male	119,353 (58.31%)	1,388 (58.72%)	362 (55.95%)	768 (59.40%)
Female	71,654 (35.00%)	831 (35.15%)	233 (36.01%)	453 (35.03%)
Not specified	13,695 (6.69%)	145 (6.13%)	52 (8.04%)	72 (5.57%)
Age (years)				
≥65	86,276 (42.15%)	1,016 (42.98%)	229 (35.39%)	600 (46.41%)
<65	72,826 (35.58%)	979 (41.41%)	278 (42.97%)	521 (40.29%)
Not specified	45,600 (22.27%)	369 (15.61%)	140 (21.64%)	172 (13.30%)
Reporter years				
2015	8,222 (4.02%)	70 (2.96%)	33 (5.10%)	19 (1.47%)
2016	18,643 (9.11%)	161 (6.81%)	39 (6.03%)	87 (6.73%)
2017	28,124 (13.74%)	277 (11.72%)	80 (12.36%)	137 (10.60%)
2018	38,222 (18.67%)	486 (20.56%)	129 (19.94%)	250 (19.33%)
2019	46,839 (22.88%)	545 (23.05%)	146 (22.57%)	325 (25.13%)
2020	47,998 (23.45%)	597 (25.25%)	172 (26.58%)	328 (25.37%)
2021Q1	16,654 (8.13%)	228 (9.65%)	48 (7.42%)	147 (11.37%)
Report countries				
United States	69,701 (34.05%)	836 (35.36%)	235 (36.32%)	426 (32.95%)
Japan	44,065 (21.52%)	703 (29.74%)	127 (19.63%)	505 (39.06%)
France	17,764 (8.68%)	166 (7.02%)	59 (9.12%)	84 (6.50%)
Other countries	73,172 (35.75%)	659 (27.88%)	226 (34.93%)	278 (21.49%)
Cancer types				
Lung cancer	69,076 (33.75%)	620 (26.23%)	185 (28.59%)	341 (26.37%)
Melanoma	46,694 (22.81%)	725 (30.67%)	204 (31.53%)	397 (30.71%)
Renal cancer	20,020 (9.78%)	357 (15.10%)	81 (12.52%)	206 (15.93%)
Gastric cancer	3,686 (1.80%)	79 (3.34%)	7 (1.08%)	67 (5.18%)
Head and neck	4,653 (2.27%)	37 (1.56%)	9 (1.39%)	19 (1.47%)
Bladder cancer	2,874 (1.40%)	37 (1.56%)	10 (1.55%)	22 (1.70%)
Colorectal cancer	2,289 (1.12%)	24 (1.02%)	9 (1.39%)	9 (0.70%)
Others	55,410 (27.07%)	485 (20.52%)	142 (22.95%)	232 (17.94%)

In Table 1, ICIs, Immune checkpoint inhibitors; ICIPI, ICI-related pancreatic injury; ICI-P, ICI-associated pancreatitis; ICI-DM, ICI-associated diabetes mellitus.

With respect to age, patients aged <65 years with pancreatic AEs had a higher reporting frequency than patients aged ≥65 years (1.34 *vs*. 1.18%). The difference between these results was significant (χ^2^ = 8.861, *p* = 0.003). Additionally, patients aged <65 years with ICI-P accounted for a larger proportion than patients aged ≥65 years (42.97 *vs*. 35.39%), and a significant difference was observed in the reporting frequency (0.38 *vs*. 0.27%, χ^2^ = 16.817, *p* < 0.001). Interestingly, no significant difference was observed in the reporting frequency by age in patients with ICI-DM (<65 years [0.72%] *vs*. ≥65 years [0.70%], χ^2^ = 0.225, *p* = 0.635).

Considering that many factors may affect the disproportionality signals, we analyzed whether concomitant drugs potentially causing pancreatitis (Supplementary Table S3) were reported and investigated overlap with other irAEs (diabetes mellitus, colitis, and hepatitis) in the cases of ICI-associated pancreatitis. In our analysis, 14.53% of patients with ICI-associated pancreatitis were exposed to concomitant drugs defined as class I drugs that may cause pancreatitis (Badalov et al., 2007). Of the 647 cases of ICI-associated pancreatitis, 4.18% were co-reported with diabetes mellitus, 22.10% were co-reported with hepatitis, and 14.53% were co-reported with colitis.

### Signal Values Associated With Different Immunotherapy Regimens

To explore the ICI-associated pancreatic AEs, we firstly compared ICIs with the full database. The signal values used to assess the association between total/class-specific ICIs and pancreatic AEs are shown in Supplementary Table S4. Generally, ICIs immunotherapy was significantly associated with the reporting frequency of pancreatic AEs (ROR_025_ = 3.30, IC_025_ = 1.71). Regarding monotherapy, the majority of pancreatic AEs were reported in patients using anti-PD-1 (58.46%), corresponding to the strongest signal (ROR_025_ = 2.97, IC_025_ = 1.56). Our study demonstrated a stronger association of ICIPI among patients who received combination therapy compared with those who received monotherapy (ROR_025_ = 5.08, IC_025_ = 2.33 *vs*. ROR_025_ = 2.84, IC_025_ = 1.50).

**FIGURE 1:**
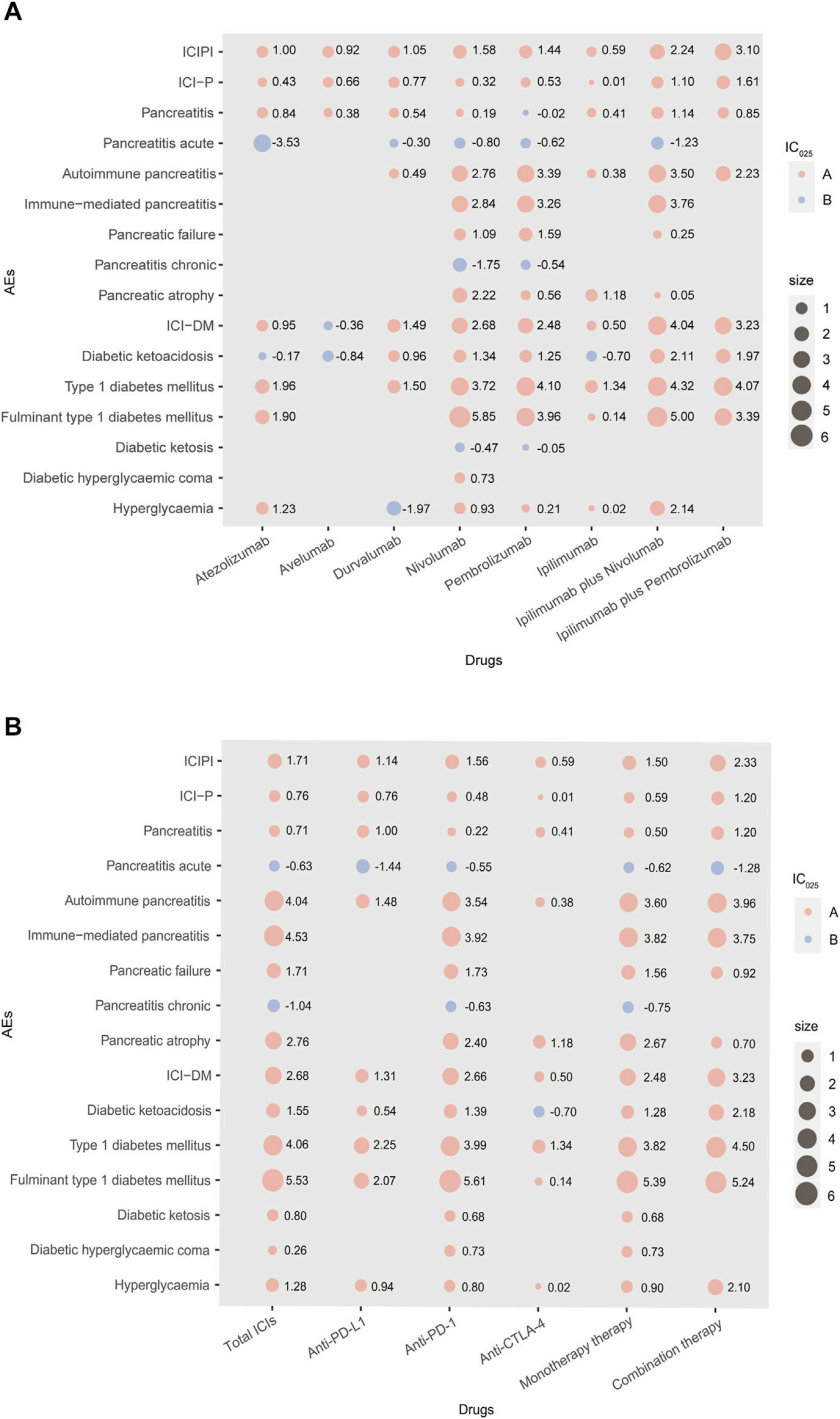
**(A,B)** Pancreatic AEs signals profiles of different ICIs strategies with all drugs as comparator. In Panel 1, AEs: adverse events; IC: information component; IC_025_: the lower end of the 95% confidence interval of IC; A: IC_025_ > 0; B: IC_025_ < 0; size: absolute value of IC_025_; ICI-P: ICI-associated pancreatitis; ICI-DM: ICI-associated diabetes mellitus; combination therapy: nivolumab plus ipilimumab, pembrolizumab plus ipilimumab.

Since cemiplimab was approved in September 2018 and is only used to treat patients with metastatic or locally advanced skin squamous cell carcinoma unsuitable for surgery or radiotherapy (Markham and Duggan, 2018), only a few reports of AEs induced by cemiplimab are available. Therefore, cemiplimab was excluded from further analysis, and the pancreatic AEs signal spectrum of different ICIs treatments is shown in Figure 1 with IC_025_ regarded as an indicator.

As shown in Figure 1, nivolumab presented the broadest spectrums among monotherapies, with a total of 10 PTs as potential signals observed. For nivolumab and pembrolizumab, there were 8 PTs in common, in which fulminant type 1 diabetes mellitus and type 1 diabetes mellitus were the two strongest signals, ranked first (IC_025_ = 5.85, IC_025_ = 3.96) and second (IC_025_ = 3.72, IC_025_ = 4.10), respectively. Overall, only one overlapping PT (pancreatitis) was observed a significant association with anti-PD-L1 therapy involving atezolizumab, avelumab and durvalumab. Regarding ipilimumab, the only anti-CTLA-4 drug included in this study, produced 6 potential signals, in which type 1 diabetes mellitus posed the strongest signal (IC_025_ = 1.34). For combination therapies, ipilimumab plus nivolumab was observed more PTs with significance than ipilimumab plus pembrolizumab, with Fulminant type 1 diabetes mellitus observed as the strongest signal (IC_025_ = 5.00). Accordingly, the signals of specific pancreatic AEs (especially ICI-P and ICI-DM) may differ in different ICIs treatments, as their PTs correspond to different signals in the same treatment.

Further analysis was performed to determine whether there was a difference between different ICIs and specific pancreatic AEs (ICI-P and ICI-DM). In our analysis, significant signals were noted between specific pancreatic AEs (pancreatitis and diabetes mellitus) and ICIs treatments (ROR_025_ = 1.71, IC_025_ = 0.76; ROR_025_ = 6.45, IC_025_ = 2.68, respectively). For ICI-P, the strongest signal among monotherapy was reported for anti-PD-L1 (ROR_025_ = 1.75, IC_025_ = 0.76), followed by anti-PD-1 (ROR_025_ = 1.42, IC_025_ = 0.48) and anti-CTLA-4 (ROR_025_ = 1.08, IC_025_ = 0.01). For ICI-DM, cases were more frequently recorded with anti-PD-1 among monotherapies (63.73%), corresponding to the strongest signal (ROR_025_ = 6.39, IC_025_ = 2.66), especially nivolumab (42.30%, ROR_025_ = 6.49, IC_025_ = 2.68). On the contrary, anti-CTLA-4 was associated with the lowest reporting frequency of ICI-DM (ROR_025_ = 1.53, IC_025_ = 0.50). Additionally, combination therapy resulted in a higher reporting frequency of both ICI-P and ICI-DM than monotherapy (ROR_025_ = 2.35, IC_025_ = 1.20 *vs*. ROR_025_ = 1.52, IC_025_ = 0.59; ROR_025_ = 9.53, IC_025_ = 3.23 *vs*. ROR_025_ = 5.63, IC_025_ = 2.48, respectively).

To assess the pancreatic AEs of ICIs further, we compared ICIs with different chemotherapies. As shown in Supplementary Figure S1, ICIs had the relatively stronger signal of ICI-P (ROR_025_ = 1.86, IC_025_ = 0.92) but the relatively weak signal of ICI-DM (ROR_025_ = 2.84, IC_025_ = 1.57), with chemotherapeutic drugs as comparators (all drugs in the database as the reference group). Meanwhile, no significant signals presented in the spectrum of pancreatic AEs disappeared. On the contrary, two more potential signals were detected, concerning chronic pancreatitis induced by anti-PD-1 (IC_025_ = 0.16) and diabetic ketoacidosis induced by anti-CTLA-4 (IC_025_ = 1.67). Although the signal intensities of some pancreatic AEs were not the same when two different comparators were selected separately, they are generally consistent in respect of the association between ICIs treatments and specific pancreatic AEs (ICI-P and ICI-DM). For example, compared with chemotherapy, anti-PD-L1 also appeared to have the strongest association with ICI-P among all monotherapies, while anti-PD-1 still seemed to be highly associated with ICI-DM. These data increase the robustness of the findings and also provide a more clinically-oriented/relevant perspective.

Since anti-PD-L1 appeared to have the strongest associations with pancreatitis, we specifically investigated whether there existed concomitant drugs and co-reported AEs in cases of pancreatitis induced by anti-PD-L1. Further analysis showed that there were only 11.46% (less than 14.53% as mentioned above) of patients with exposure to concomitant drugs potentially causing pancreatitis. The overlap of irAEs (diabetes mellitus, colitis, and hepatitis) of cases of pancreatitis induced by anti-PD-L1 was roughly similar to those of pancreatitis induced by total ICIs.

### Time to Onset of ICI-Associated Pancreatic Adverse Effects

**FIGURE 2:**
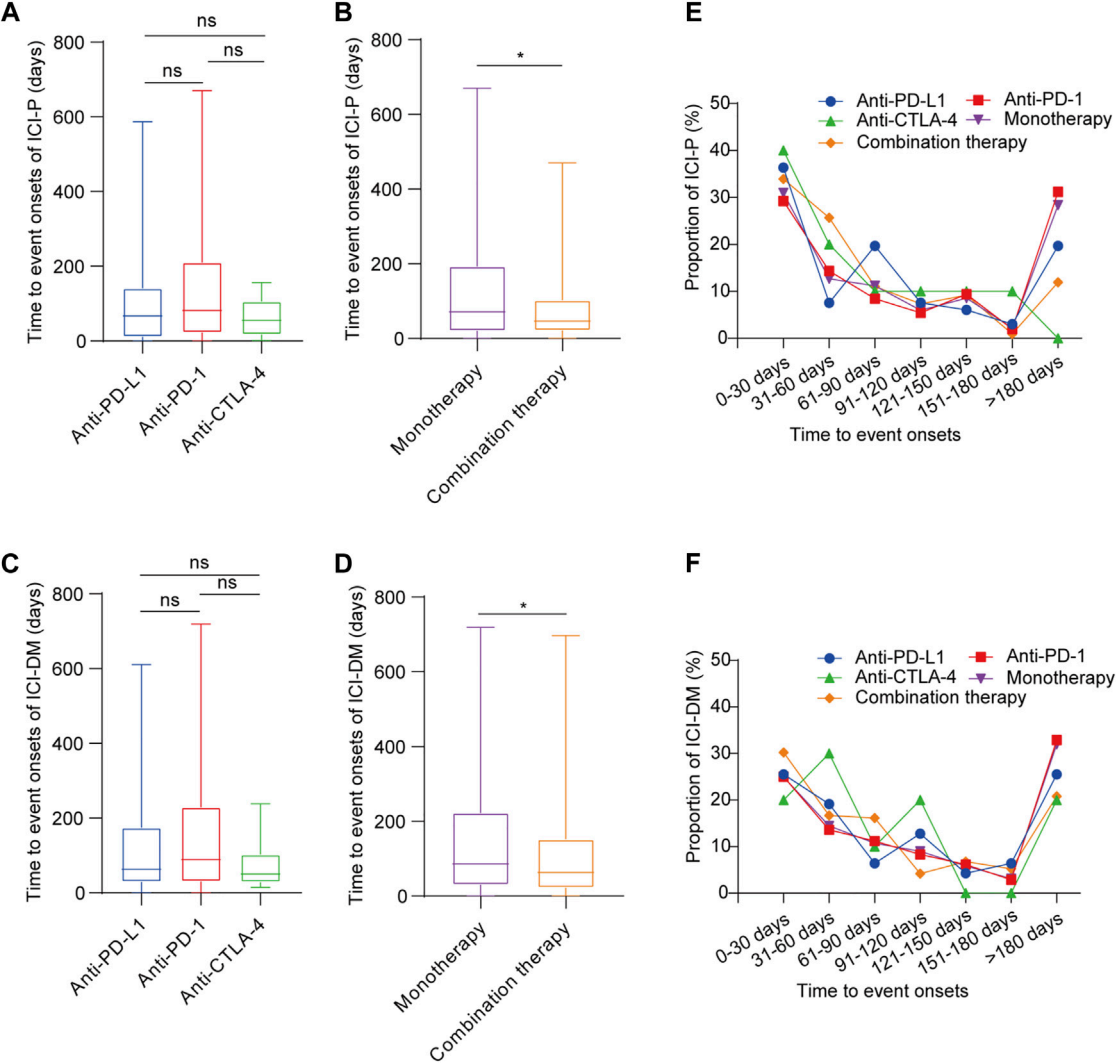
Time to onset of ICI-associated pancreatic AEs following immune checkpoint inhibitor treatments. **(A,B)** Time to onset of ICI-P following immune checkpoint inhibitor treatments. Kruskal-Wallis test or Mann-Whitney test, *p < 0.05. **(C,D)** Time to onset of ICI-DM following immune checkpoint inhibitor treatments. Kruskal-Wallis test or Mann-Whitney test, *p < 0.05. **(E)** The distribution of time to onset of pancreatitis following immune checkpoint inhibitor treatments. **(F)** The distribution of time to onset of diabetes mellitus following immune checkpoint inhibitor treatments.

Overall, 1,397 ICI-associated pancreatic AEs onset times were reported, and the median time to onset (TTO) of ICIPI was 66 (interquartile range [IQR] 26–176) days. Regarding ICI-P, the median TTO was 55 (IQR 20–90.75) days for anti-CTLA-4, 67 (IQR 11.25–138) days for anti-PD-L1, and 81.5 (IQR 23.25–209.75) days for anti-PD-1. Surprisingly, there was no statistical difference in the onset time of ICI-P among monotherapies (Figure 2A). The TTO of ICI-P following combination therapy appeared to have an earlier onset time when compared with monotherapy (47 days [IQR 22.5–102] *vs*. 71.5 days [IQR 20.25–191]), with a significant difference (Figure 2B). Regarding ICI-DM, the data shows that the shortest median TTO was 50 (IQR 31–100) days for anti-CTLA-4, the longest was 89 (IQR 30–228.5) days for anti-PD-1, and the median onset time was 63(IQR 30–169) days for anti-PD-L1. Similarly, we found no statistical difference in the onset time of ICI-DM among ICIs monotherapy groups (Figure 2C). The TTO of combination therapy was also significantly earlier than that of monotherapy (63 days [IQR 22.25–150.75] *vs*. 86 days [IQR 30–222]), with a significant difference (Figure 2D). Notably, the TTO of ICI-DM was later than that of ICI-P (76 days [IQR 28–197.5] *vs*. 62.5 days [IQR 21–147]), and the difference between these results was significant.

Among ICIPI cases, 29.28% of them occurred within 1 month after ICIs treatments, and the proportion gradually decreased within 2–6 months, while the proportion increased when the onset time extended to more than 6 months after ICIs treatment except for anti-CTLA-4 (Supplementary Figure S2). The distribution of TTO of ICI-P cases (Figure 2E) and ICI-DM cases (Figure 2F) was roughly the same as that of total pancreatic AE cases following ICIs treatments except for anti-CTLA-4.

### Fatality and Hospitalization Proportion due to ICI-Associated Pancreatic Adverse Effects

**FIGURE 3:**
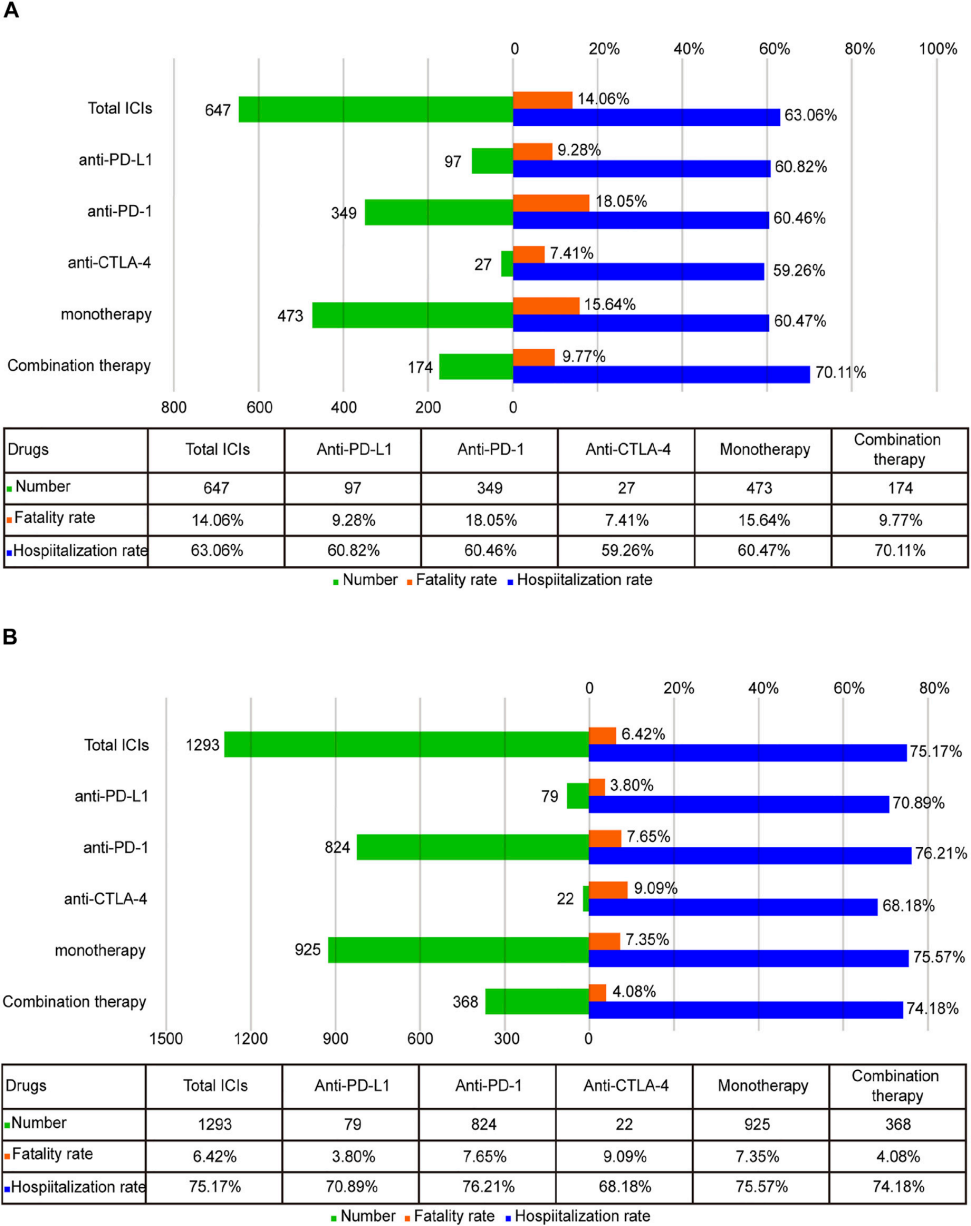
**(A)** The number of reports, hospitalization, and fatality proportions for ICI-associated pancreatitis. **(B)** The number of reports, hospitalization, and fatality proportions for ICI-associated diabetes mellitus.

To better determine the prognosis of ICI-associated pancreatic AEs, the fatality and hospitalization proportions were assessed. In general, the fatality proportion of the ICIPI was 9.90% (Supplementary Figure S3). For ICI-P, the highest fatality proportion was reported for anti-PD-1 among all monotherapies (18.05%, 63 deaths of 349 cases), followed by anti-PD-L1 (9.28%, 9 deaths of 97 cases), and the lowest was anti-CTLA-4 (7.41%, 2 deaths of 27 cases). Nevertheless, no significant difference was found in the fatality proportion across different ICIs monotherapies (χ^2^ = 5.899, *p* = 0.052). Notably, the fatality proportion in monotherapy was higher than that in combination therapy (15.64 *vs* 9.77%, χ^2^ = 3.666, *p* = 0.056). Hospitalization (63.06%) was the most common outcome in ICI-P (Figure 3A). Interestingly, there was no statistical significance observed in hospitalization proportion in patients treated with monotherapies (χ^2^ = 0.022, *p* = 0.989). Importantly, the hospitalization proportion in combination therapy was higher than that in all monotherapies (70.11 *vs*. 60.47%, χ^2^ = 4.954, *p* = 0.026). Further analysis was conducted to assess the prognosis of patients with ICI-DM (Figure 3B). Among all monotherapies, there was no significant difference in the fatality proportion of patients with ICI-DM (χ^2^ = 1.667, *p* = 0.434), nor was the statistical significance observed in hospitalization proportion (χ^2^ = 1.774, *p* = 0.412). The fatality of combination therapy in ICI-DM was significantly lower than that of monotherapy (4.08 *vs*. 7.35%, χ^2^ = 4.719, *p* = 0.03); however, no significant difference was observed in hospitalization proportion (74.18 *vs*. 75.57%, χ^2^ = 0.303, *p* = 0.582).

## Discussion

A number of ICIs pharmacovigilance studies have been conducted to identify potential associations between ICIs regimens and adverse events from the FAERS database using disproportionality measures, the majority of which focused on specific irAEs involving specifically targeted organs, such as cardiotoxicity, colitis, and renal toxicity (Chen et al., 2020; Hu et al., 2020; Chen et al., 2021a). As previously reported, each ICI regimen had different characteristics of irAEs (Chen et al., 2021b). For example, gastrointestinal (colitis) and endocrine toxicity (hypophysitis, adrenal insufficiency, hypopituitarism) were more preferentially reported with anti-CTLA-4, whereas thyroid dysfunction, pneumonitis and myocarditis were more frequently recorded with anti-PD-1/PD-L1 (Raschi et al., 2020). With the rapid development of ICIs indications in recent years, the awareness of ICI-associated pancreatic AEs has grown. However, the relationship between different ICIs and pancreatic AEs has yet to be adequately examined. Therefore, we investigated ICI-associated pancreatic AEs from the FAERS database to identify and evaluate the relationship between ICIs and pancreatic AEs, which will serve as a reference for future prevention and therapy. Our findings are as follows:

In total, 2,364 cases of ICIPI were included in the study, which we believe is the largest collection of cases of ICIPI to date. In our analysis, the number of pancreatic AEs following ICIs therapy has gradually increased every year, reflecting the increased prevalence of ICIs use in oncology treatment. Remarkably, ICIPI seemed to predominately affect males, and melanoma was the most common type of cancer associated with ICIPI. This result was consistent with the findings of a previous report (Abu-Sbeih et al., 2019). Nevertheless, it is noteworthy that female patients were often excluded from clinical trials and not recommended for using ICIs in most cases, given that they have a higher tendency to trigger autoimmune diseases than male patients (Quintero et al., 2012; Conforti et al., 2018; Ma et al., 2021). Therefore, further analysis was performed to investigate the effect of sex on the reporting frequency of pancreatic AEs following ICIs treatment. In general, patients with ICIPI had similar reporting frequencies between male and female patients. Similarly, no significant difference was observed in the reporting frequency according to sex in patients with ICI-P or ICI-DM. The analysis of the association between sex and pancreatic AEs corroborated the results of a previous retrospective study, that is, there was no significant difference in irAEs between male and female patients (Jing et al., 2021).

Our study also noted that patients aged <65 years with ICI-P had a higher reporting frequency than those aged ≥65 years. Regarding ICI-DM, for which no significant difference has been observed in the incidence by age (Liu et al., 2020), statistical significance was also not found in the reporting frequency in our study.

Indeed, the effect of age difference on irAEs is controversial, 1) as some previous studies have reported that elderly patients showed slightly higher incidences of irAEs (Baldini et al., 2020), 2) some indicated that age distribution differed in different profiles of irAEs (Paderi et al., 2021), and 3) some found that age was not associated with irAEs (Gomes et al., 2021; Noseda et al., 2021). To some extent, our results are consistent with the second finding. Based on the tremendous records in FAERS, our study may provide some useful clinical evidence of the associations between age and irAEs. More attention should be focused on the age differences in patients with irAEs in future studies.

Notably, significant signals were detected between pancreatic AEs and all ICIs treatments. Conspicuously, we observed that signals of specific pancreatic AEs (ICI-P and ICI-DM) caused by anti-PD-L1, anti-PD-1, and anti-CTLA-4 differed. In our analysis, anti-PD-L1 was observed to have the strongest association with ICI-P among all monotherapies, which was in line with the result of a previous study (Reese et al., 2020). Additionally, co-reported AEs (diabetes mellitus, colitis and hepatitis) and concomitant drugs potentially causing pancreatitis had little effect on the disproportionality signal of pancreatitis induced by anti-PD-L1 when compared with total ICIs. Moreover, patients with pancreatitis induced by anti-PD-L1 were less exposed to concomitant drugs at risk of causing pancreatitis. However, the association between different ICIs and pancreatitis remains controversial, as some other studies have demonstrated that both anti-CTLA4 alone and combination treatments with nivolumab and ipilimumab are associated with a higher incidence of pancreatitis than anti-PD-1/PD-L1 alone (Su et al., 2018; George et al., 2019; Bai et al., 2021). Consequently, prospective studies are warranted to further investigate and ascertain the true association between pancreatitis and different ICIs treatments.

Regarding ICI-DM, a higher reporting frequency of diabetes mellitus was observed in all ICIs treatments versus all drugs or chemotherapy. It appears that anti-PD-1 was likely to result in more ICI-DM. Through retrospective clinical research and systematic reviews, prior studies have concluded that ICI-DM frequently occurs in the setting of exposure to anti-PD-1/PD-L1, either alone or in combination with other immunotherapies, and emerged infrequently after anti-CTLA-4 (Stamatouli et al., 2018; Marchand et al., 2019; Quandt et al., 2020). Additionally, preclinical studies support our results, suggesting that it is a PD-1 inhibitor rather than a CTLA-4 inhibitor that rapidly induced diabetes in adult mice (Ansari et al., 2003). Notably, our study demonstrated that both ICI-P and ICI-DM showed stronger associations with combination therapy when compared with monotherapy, which was consistent with the findings of previous studies (Abu-Sbeih et al., 2019; Liu et al., 2020; Porcu et al., 2020; Liu et al., 2021). In fact, in addition to pancreatic AEs, combination therapy was reported to have a higher risk of developing other irAEs relative to monotherapy, notwithstanding the impressive activity in multiple cancers (Grimaldi et al., 2016), which should be fully recognized.

To date, the exact mechanism of the development of irAEs has yet to be elucidated, and potential mechanisms may include enhanced T cells activity against antigens present on tumor and normal tissues; increased concentrations of pre-existing autoimmune antibodies; increased levels of inflammatory cytokines, CTLA-4 antibody directly binding to normal tissues expressing CTLA-4 (such as pituitary gland), thereby promoting the enhancement of complement-mediated inflammatory response (Passat et al., 2018). Differences in the association between different ICIs and pancreatic AEs may be related to modulation of different T-cell colonies and cytokines, as previous studies have demonstrated that anti-PD-1 diabetes is associated with the predominance of exhausted CD8 cells producing IFN-γ expression, whereas anti–CTLA-4 colitis has demonstrated the predominance of CD4 cells and tumor necrosis factor-alpha expression (Reese et al., 2020; Mourad et al., 2021). In addition, anti-PD-1 may augment Th1 and Th17 responsiveness and inhibit Th2 responsiveness, while anti-CTLA-4 was observed to potentiate IL-2 only and no modulation of other Th1/Th2/Th17 effector cytokines was found with this antibody (Dulos et al., 2012). Nonetheless, the precise mechanism underlying ICI-P and ICI-DM and the relationship with each other remains to be elucidated.

In the time-to-event analysis, the median time from drug initiation to the onset of ICIPI in this study was 66 (IQR 26–176) days. Although the majority of ICIPI cases were observed within the first 6 months, it is important to note that the onset time of pancreatic AEs may extend to 6 months or even longer (2 years) after ICIs treatment. ICI-P is one of the most common pancreatic AEs associated with ICIs treatments, and further analysis showed that the median TTO of ICI-P was much earlier than the previous observation in a retrospective study (Abu-Sbeih et al., 2019). Based on the median onset time, it seemed that anti-CTLA-4 led to ICI-P in a shorter time relative to anti-PD-1/PD-L1, which was concordant with a previous study showing that irAEs tend to have slightly later onset following anti-PD-1 treatment than anti-CTLA-4 treatment (Sosa et al., 2018). For ICI-DM, it has been reported that the median time of diagnosis is between 7 and 17 weeks (Clotman et al., 2018; Stamatouli et al., 2018; Wright et al., 2018; Akturk et al., 2019). Evidence from our study supports this finding. Moreover, both ICI-P and ICI-DM occurred earlier when two ICIs were combined than when monotherapy was administered. Interestingly, the onset time of ICI-DM was later than that of ICI-P. In a previous systematic review, it has been reported that the happening of diabetes can also be a complication of pancreatitis caused by ICIs treatments (Marchand et al., 2019), but this remains to be verified.

The prognosis (especially fatality proportion and hospitalization) of ICI-associated pancreatic AEs was studied in detail. For ICI-P, anti-PD-1 led to the highest fatality among monotherapies, which had not been reported in previous studies. Interestingly, patients with ICI-P induced by combination therapy suffered from a significantly higher hospitalization proportion than those with ICI-P induced by monotherapy, while the former suffered from a lower fatality proportion than the latter. Similarly, monotherapy resulted in a higher fatality proportion than combination therapy in patients with ICI-DM. These findings seem to contradict the results of previous studies showing that irAEs occurred in combination therapy with a higher incidence rate and slightly higher severity (Grimaldi et al., 2016; Hao et al., 2017). Several reasons may account for this discrepancy. First, the data extracted from the real world is different from clinical trials, wherein strict patient selection criteria are required and the balance of sex, age, and health status needs to be taken into consideration. In the real world, combination drugs are more commonly administered in relatively young patients to overcome possible AEs. As demonstrated in our study, patients treated with nivolumab plus ipilimumab were younger than those treated with nivolumab alone (*p* < 0.001). Second, doses of combination therapy were usually reduced, in contrast to monotherapy (Larkin et al., 2015). Third, combination therapy was found to have a stronger association with pancreatic AEs, and hospitalization caused by ICI-associated pancreatic AEs in combination therapy was higher than monotherapy, indicating the possibility of more systematic monitoring as well as specialized care in combination therapy.

Although these results largely correlate with the previous literature, there are several limitations to our study. First, detailed information on clinical data, which might contribute to a more comprehensive conclusion about the association between specific AEs and ICIs, was unavailable in the FAERS database. Second, the FAERS database is a spontaneous reporting system with missing data, data duplication, nonuniform data format, and reporting bias (e.g., under-reporting and selective reporting) as well as geographical bias. No incidence of AE could be calculated from FAERS because of the lack of a denominator and under-reporting. Additionally, fatality rates could not be calculated since there are no total exposure data, also considering that death can be also caused by the underlying disease, co-reported irAEs, and other events. Third, a causal relationship cannot be directly proven, as this was a retrospective study. Finally, data involving several drugs and/or several AEs were extracted as a unit of combination drug-AE pairs rather than reports, which may lead to bias in the results of pharmacovigilance analysis.

Notwithstanding these limitations, our study provided a signal profile of ICI-associated pancreatic AEs, which may provide valuable evidence for further research and clinical practice in this field. However, it is important to point out that any results generated through pharmacovigilance databases should be validated by prospective studies.

## Conclusion

Overall, ICIs were significantly associated with pancreatic AEs, including ICI-P and ICI-DM. Compared with monotherapy, combination therapy showed stronger associations with both pancreatitis and diabetes mellitus, but lower fatality proportion. Clinicians should be aware of the possibility that ICIs may lead to pancreatic AEs despite their rare incidence, and it is necessary to inform patients of these potential toxicities before ICIs medications are administered.

## Data Availability

Publicly available datasets were analyzed in this study. This data can be found here: https://www.fda.gov/drugs/questions-and-answers-fdas-adverse-event-reporting-system-faers/fda-adverse-event-reporting-system-faers-public-dashboard.

## Abbreviations

AE, adverse event; CTLA-4, cytotoxic T lymphocyte antigen-4; FAERS, Food and Drug Administration Adverse Event Reporting System; ICIs, immune checkpoint inhibitors; irAEs, immune-related adverse events; ICIPI, ICI-related pancreatic injury; ICI-DM, ICI-associated diabetes mellitus; ICI-P, ICI-associated pancreatitis; IC, information component; NSCLC, non-small cell lung cancer; PD-1, programmed cell death protein; PTs, preferred terms; ROR, reporting odds ratio; TTO, time to onset.
