# Supplementary material for: Pancreatic Adverse Events Associated With Immune Checkpoint Inhibitors: A Large-Scale Pharmacovigilance Analysis
Source: Front Pharmacol. 2022 Apr 1;13:817662. doi: 10.3389/fphar.2022.817662 (PMC9012537; doi:10.3389/fphar.2022.817662)
Supplement: Supplementary file 1 [file DataSheet1.zip › Supplemenary Material.docx]

1. **Supplementary Figure**

Supplementary Figure S1. (A) and (B) Pancreatic AEs signals profiles of different ICI strategies with chemotherapeutic drugs as comparator. AEs: adverse events; IC: information component; IC_025_: the lower end of the 95% confidence interval of IC; A: IC_025_ >0; B: IC_025_ <0; size: absolute value of IC_025_; ICI-P: ICI-associated pancreatitis; ICI-DM: ICI-associated diabetes mellitus; combination therapy: Nivolumab plus Ipilimumab, Pembrolizumab plus Ipilimumab.

Supplementary Figure S2. The distribution of time to onset of ICIPI following immune checkpoint inhibitor treatments. ICIPI: ICI-related pancreatic injury.

Supplementary Figure S3. The number of reports, hospitalization, and fatality proportions for ICI-associated pancreatic adverse events.

1. **Supplementary Table**

Supplementary Table S1. The preferred terms (PTs) of pancreatic adverse events.

| ICIPI | PTs | MedDRA Code |
| --- | --- | --- |
| ICI-P | Pancreatitis | 10033645 |
|  | Autoimmune pancreatitis | 10069002 |
|  | Pancreatitis acute | 10033647 |
|  | Immune-mediated pancreatitis | 10083072 |
|  | Pancreatitis chronic | 10033649 |
|  | Pancreatitis necrotising | 10033654 |
|  | Oedematous pancreatitis | 10052400 |
|  | Pancreatitis haemorrhagic | 10033650 |
|  | Pancreatic pseudocyst | 10033635 |
|  | Pancreatic enlargement | 10055024 |
|  | pancreatic failure | 10079281 |
|  | Pancreatic atrophy | 10033603 |
| ICI-DM | Diabetic ketoacidosis | 10012671 |
|  | Type 1 diabetes mellitus | 10067584 |
|  | Fulminant type 1 diabetes mellitus | 10072628 |
|  | Diabetic hyperglycaemic coma | 10012668 |
|  | Diabetic ketosis | 10012673 |
|  | Hyperglycaemia | 10020639 |

Supplementary Table S2. Standard drug name and original drug name of chemotherapy in FAERS database.

| **Standard drug name** | **Original drug name in FARES database** |
| --- | --- |
| Cisplatin | "CISPLATIN.", "CISPLATINE", "CISPLATIN (Manufacturer unknown)", "CISPLATIN (Manufacturer unknown"), "CISPLATINUM" ... |
| Carboplatin | "CARBOPLATIN.", "PARAPLATIN", "CARBOPLATINE", "Carboplatine Hospira", "CARBOPLATIN (Manufacturer)", "CARBOPLATIN (Manufacturer Unknown)"… |
| Nedaplatin | "NEDAPLATIN", "Nedaplatin", "AQUPLA", "LUBEI (NEDAPLATIN)", "JIE BAI SHU", "nedaplatin" |
| Dicycloplatin | "BLINDED PLACEBO", "BLINDED NO TREATMENT RECEIVED", "BLINDED Placebo", "BLINDED THERAPY", "INVESTIGATIONAL DRUG"… |
| Oxaliplatin | "OXALIPLATIN.", "ELOXATIN", "ELPLAT", "OXALIPLATINE", "OXALIPLATIN (Manufacturer)", "OXALIPLATIN (Manufacturer unknown)", "OXALIPLATIN ACCORD"… |
| Lobaplatin  Capecitabine | "LOBAPLATIN", "Lobaplatin", "LOBAPLANTIN"  "XELODA", "CAPECITABINE.", "CAPECITABINE TAB 500MG", "CAPECITABINE TAB 500 MG", "CAPECITABINE 500MG"… |
| Paclitaxel | "PACLITAXEL.", "ABRAXANE", "TAXOL", "PACLITAXEL/PACLITAXEL LIPOSOME", "PACLITAXEL (Manufacturer)", "NAB-PACLITAXEL" |
| Docetaxel | "TAXOTERE", "DOCETAXEL.", "Docetaxel Accord", "DOCETAXEL ACCORD", "DOCETAXEL SANDOZ", "Docetaxel Hospira", "DOCETAXEL HOSPIRA"… |
| Uracil  Tegafur | "URACIL"， "uracil"， "Uracil"  "TEGAFUR", "Tegafur", "TEGAFUR/TEGAFUR SODIUM", "TEGAFUR URACIL", "FTORAFUR"… |
| Etoposide | "ETOPOSIDE.", "ETOPOSIDE (VP-16)", "VP-16", "VEPESID", "ETOPOSIDE (Manufacturer Unknown)", "Etoposide (VP-16)", "ETOPOSIDE (Manufacturer Unknown"… |
| Dacarbazine | "DACARBAZINE.", "DETICENE", "DACARBAZINE (Manufacturer unknown)"，"DACARBAZINE MEDAC", "DTIC", "DACARBACIN"… |
| Temozolomide | "TEMOZOLOMIDE.", "TEMODAR", "TEMODAL", "TEMOZOLOMIDE CAP 100MG"， "TEMOZOLOMIDE CAP 140MG"，"TEMOZOLOMIDE 100MG CAP"， "TEMOZOLOMIDE 140MG CAP"， |
| Pemetrexed | "PEMETREXED."， "PEMETREXED"， "Pemetrexed"，"PEMETREXED SODIUM HYDRATE"， "PEMETREXED (Manufacturer U"， "Pemetrexed (Unknown)"， |
| Gemcitabine | "GEMCITABINE"，"GEMCITABINE/GEMCITABINE HYDROCHLORIDE"， "GEMCITABINE/GEMCITABINE HY"， "GEMCITABINE/GEMCITABINE HYDROCH"， "GEMCITABINE SANDOZ" |
| Sorafenib | "NEXAVAR"， "SORAFENIB"，"SORAFENIB (RAF KINASE INHIBITOR)"， "SORAFENIB (RAF KINASE INHIBITOR"， "SORAFENIB (RAF KINASE INHI"， "Sorafenib"， "sorafenib"， |
| Fluorouracil | "FLUOROURACIL."， "5-FU"， "5-FLUOROURACIL"， "5 FU"， "FLUOROURACILE"， "5-FU /00098801/"， "5-FLUOROURACIL (5-FU)"， "EFUDEX"， |
| Irinotecan | "IRINOTECAN"， "Irinotecan"， "Campto"， "CAMPTO"， "IRINOTECAN (Manufacturer u"， "CPT-11"， "irinotecan"， "IRINOTECAN (Manufacturer unknown)"， "IRINOTECAN HOSPIRA" |
| Docetaxel* | "DOCETAXEL."， "Docetaxel Accord"， "DOCETAXEL ACCORD"， "DOCETAXEL SANDOZ"， "Docetaxel Hospira"， "DOCETAXEL HOSPIRA"， |
| Doxorubicin | DOXORUBICIN"，"Doxorubicin"，"doxorubicin"，"DOXORUBICINE"， "HYDROXYDAUNORUBICIN"， "DOXORUBICINE TEVA"， "DOXORUBICIN (Manufacturer Unknown)"， "MYOCET"， |
| Epirubicin | EPIRUBICIN"， "Epirubicin"， "Pharmorubicin"， "epirubicin"， "EPIRUBICINE"， "EPIRUBICIN (Manufacturer unknown)"， "Epirubicin (Unknown)"， |
| Methotrexate | "METHOTREXATE."， "METHOTREXATE"， "METHOTREXATE (TRADE NAME UNKNOWN)"， "METHOTREXATE (TRADE NAME U"， "OTREXUP" |
| Pirarubicin  Ramosetron | PIRARUBICIN"， "PINORUBIN"， "Pirarubicin"， "pirarubicin"，  "NASEA"， "IRRIBOW"， "RAMOSETRON HYDROCHLORIDE"， "RAMOSETRON"， "Nasea"， "NASEA OD"， "Ramosetron hydrochloride"， |
| Mitomycin | "MITOMYCIN."， "MITOMYCIN C"， "AMETYCINE"， "MITOMYCINE"， "MITOMYCIN INJ 40MG" |
| Bleomycin | "BLEOMYCIN"， "BLEOMYCIN SULFATE"， "BLEOMYCINE"， "BLEOMYCIN SULPHATE"， "BLEOMYCINE BELLON"， "BLEOMYCIN ^BAXTER^"， |
| Mitoxantrone | "MITOXANTRONE"， "MITOXANTRONE HCL"， "NOVANTRONE"， "MITOXANTRONE HYDROCHLORIDE."， |
| Vindesine | VINCRISTINE"， "Vincristine"， "vincristine"， "VINCRISTIN"， "VINCRISTINE HOSPIRA"， |
| Idarubicin | "IDARUBICIN"， "Idarubicin"， "Zavedos"， "idarubicin"， "ZAVEDOS"， "IDARUBICINE"， "EXTERNAL-IDARUBICIN"， "idarubicine"， |
| Cyclophosphamide | "CYCLOPHOSPHAMIDE."， "NEOSAR"， "CYTOXAN"， "ENDOXAN"， "PROCYTOX" |
| Ifosfamide | "IFOSFAMIDE."， "HOLOXAN"， "IFEX"， "IFOSFAMIDE EG"， "IFOSFAMIDE (Manufacturer unknown)" |
| Cytarabine | "CYTARABINE."， "ARACYTINE"， "ARA-C" "Aracytine"， "CYTARABINE/CYTARABINE HYDR" |
| Bortezomib | VELCADE"， "BORTEZOMIB"， "BORTEZOMIB."， "Bortezomib"， "bortezomib"， "BORTEZOMIBE" |
| Adriamycin | "ADRIAMYCIN"， "DOXORUBICIN HYDROCHLORIDE."， "DOXIL"， "DOXORUBICIN HCL"， "CAELYX" |
| Vinblastine | "VINBLASTINE"， "Vinblastine"， "VINBLASTIN"， "vinblastine"， "VINBLASTINE (manufacturer unkno"， "Vinblastin"， |
| Etoposide | "ETOPOSIDE."， "ETOPOSIDE (VP-16)"， "VP-16"， "VEPESID"，"ETOPOSIDE (Manufacturer Unknown)"， "Etoposide (VP-16)"， |
| Teniposide | "TENIPOSIDE."， "VUMON"， "VM 26"， "TENIPOSIDE (VM-26)" |

Supplementary Table S3. Summary of Class I drugs potentially causing acute pancreatitis.

| **Ia** | **Ib** |
| --- | --- |
| α-methyldopa | All-trans-retinoic acid |
| Azodisalicylate | Amiodarone |
| Bezafibrate | Azathioprine |
| Cannabis | Clomiphene |
| Carbimazole | Dexamethasone |
| Codeine | Ifosfamide |
| Cytosine | Lamivudine |
| Arabinoside | Losartan |
| Dapsone | Lynesterol/metho-xyethinylestradiol |
| Enalapril | 6- MP |
| Furosemide | Meglumine |
| Isoniazid | Methimazole |
| Mesalamine | Nelfinavir |
| Metronidazole | Norethindronate/mestranol |
| Pentamidine | Omeprazole |
| Pravastatin | Premarin |
| Procainamide | Sulfamethazole |
| Pyritonol | Trimethoprim |
| Simvastatin |  |
| Stibogluconate |  |
| Sulfamethoxazole |  |
| Sulindac |  |
| Tetracycline |  |
| Valproic acid |  |

Supplementary Table S4. The associations of pancreatic AEs with different immunotherapy treatments.

| Drug | N | ROR | **ROR_025_** | ROR_975_ | IC | **IC_025_** | IC_975_ |
| --- | --- | --- | --- | --- | --- | --- | --- |
| Total ICIs | 2364 | 3.44 | **3.30** | 3.59 | 1.78 | **1.71** | 1.83 |
| Monotherapy | 1677 | 2.98 | **2.84** | 3.13 | 1.58 | **1.50** | 1.64 |
| Anti-PD-L1 | 228 | 2.57 | **2.25** | 2.93 | 1.36 | **1.14** | 1.52 |
| Atezolizumab | 150 | 2.40 | **2.05** | 2.82 | 1.27 | **1.00** | 1.46 |
| Avelumab | 16 | 3.38 | **2.07** | 5.54 | 1.76 | **0.92** | 2.34 |
| Durvalumab | 62 | 2.78 | **2.16** | 3.57 | 1.47 | **1.05** | 1.78 |
| Anti-PD-1 | 1382 | 3.14 | **2.97** | 3.31 | 1.65 | **1.56** | 1.71 |
| Nivolumab | 906 | 3.24 | **3.03** | 3.46 | 1.69 | **1.58** | 1.77 |
| Pembrolizumab | 472 | 3.02 | **2.76** | 3.31 | 1.60 | **1.44** | 1.71 |
| Cemiplimab | 4 | 0.88 | 0.33 | 2.34 | -0.19 | -1.96 | 0.89 |
| Anti-CTLA-4 | 67 | 1.99 | **1.57** | 2.53 | 0.99 | **0.59** | 1.29 |
| Ipilimumab | 67 | 1.99 | **1.57** | 2.53 | 0.99 | **0.59** | 1.29 |
| Combination therapy | 687 | 5.48 | **5.08** | 5.92 | 2.46 | **2.33** | 2.55 |
| Nivolumab plus Ipilimumab | 625 | 5.19 | **4.80** | 5.62 | 2.38 | **2.24** | 2.47 |
| Pembrolizumab plus Ipilimumab | 62 | 11.50 | **8.92** | 14.83 | 3.52 | **3.10** | 3.83 |

In Supplementary Table S4, bold text denotes significant signals. N: numbers of records. ROR_025_: the lower end of the 95% confidence interval of ROR. ROR_975_: the upper end of the 95% confidence interval of ROR. IC_025_: the lower end of the 95% confidence interval of IC. IC_975_: the upper end of the 95% confidence interval of IC.
