# Supplementary figures and images for: Pancreatic Adverse Events Associated With Immune Checkpoint Inhibitors: A Large-Scale Pharmacovigilance Analysis
Source: Front Pharmacol. 2022 Apr 1;13:817662. doi: 10.3389/fphar.2022.817662 (PMC9012537; doi:10.3389/fphar.2022.817662)

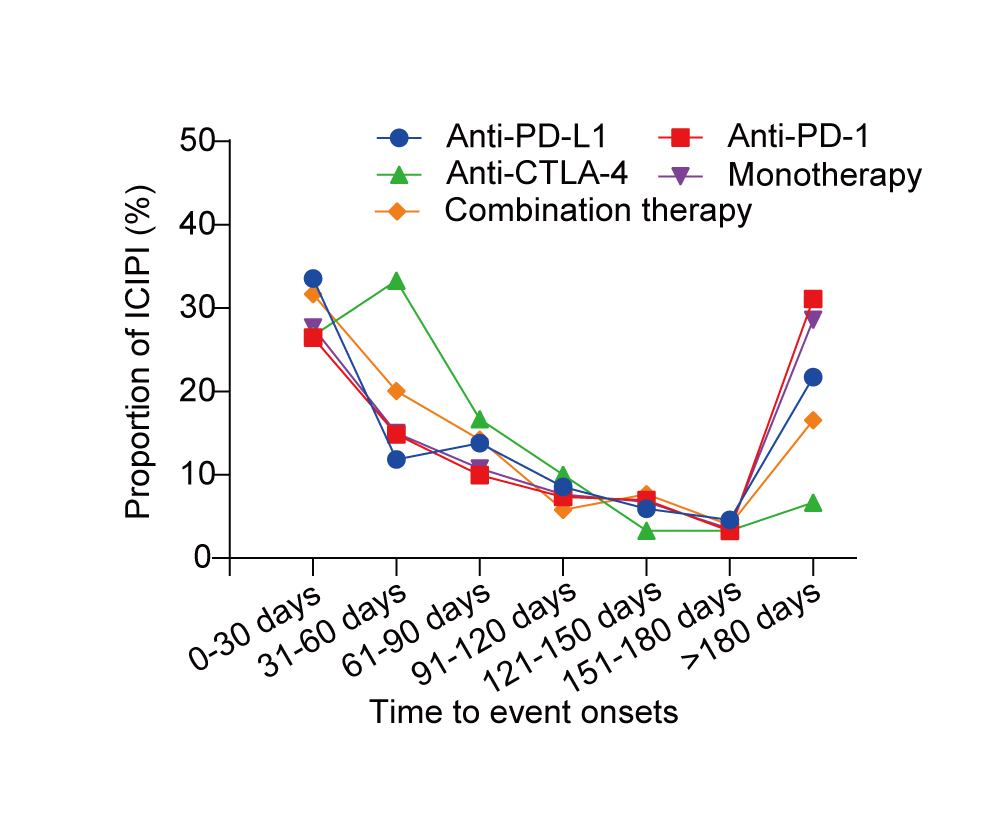

Supplement: Supplementary file 1 [file DataSheet1.zip › Supplementary Figure S2.tif]

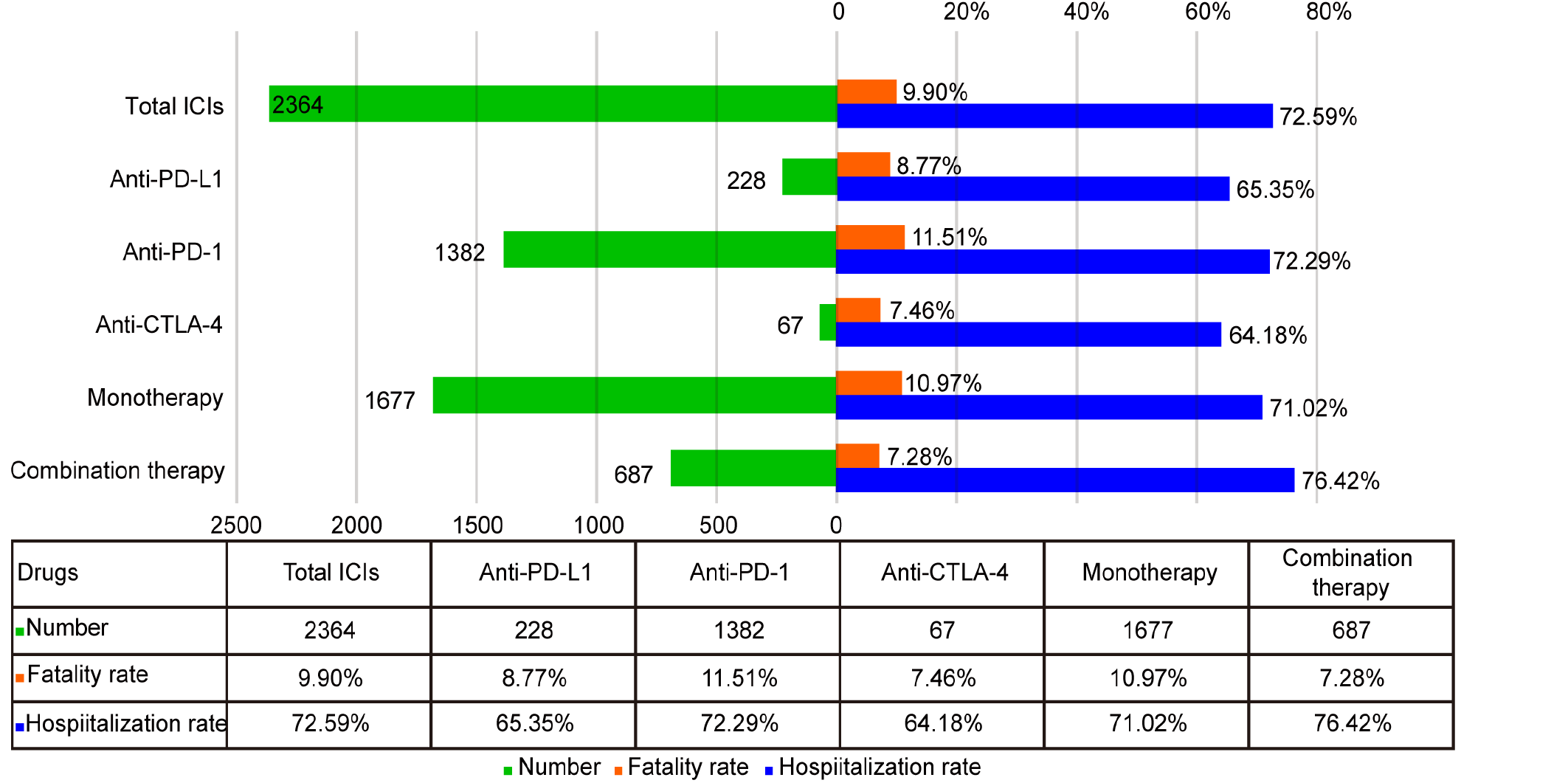

Supplement: Supplementary file 1 [file DataSheet1.zip › Supplementary Figure S3.tif]

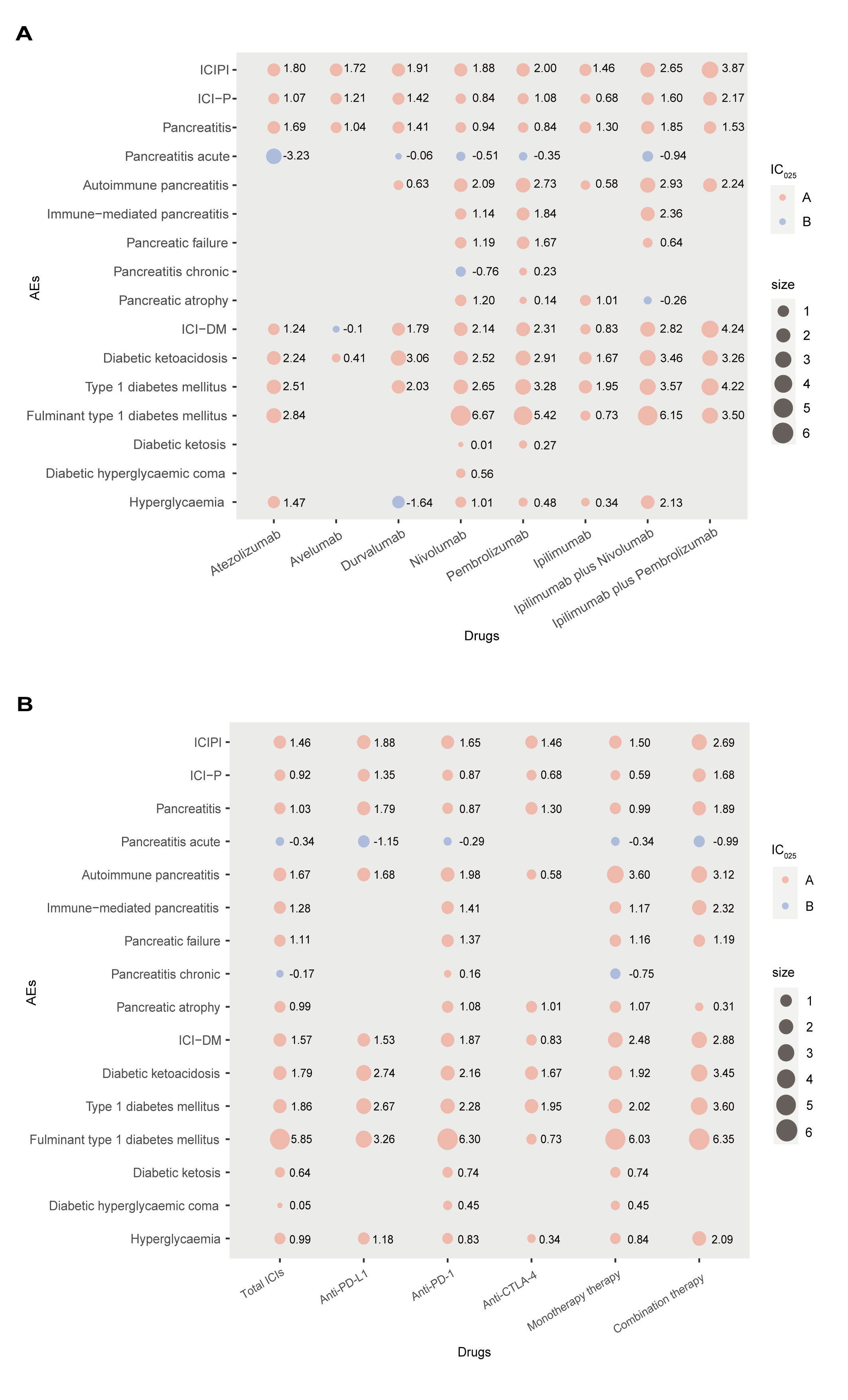

Supplement: Supplementary file 1 [file DataSheet1.zip › Supplementary Figure S1.tif]
